# ER-Negative Endometrial Cancers: An Evolving Diagnostic Category with Major Clinical Implications

**DOI:** 10.3390/cancers18050773

**Published:** 2026-02-27

**Authors:** Rujia Fan, Xiaowei Wei, Jayanthi Lea, Huiting Zhu, Wenxin Zheng

**Affiliations:** 1 Department of Obstetrics and Gynecology, Henan Provincial People’s Hospital, Zhengzhou University People’s Hospital, Henan University People’s Hospital, Zhengzhou 450003, China; fanrujia@zzu.edu.cn; 2 Department of Pathology, University of Texas Southwestern Medical Center, Dallas, TX 75390, USA; kele1996@sjtu.edu.cn; 3 Department of Obstetrics and Gynecology, Shanghai Jiao Tong University Affiliated Sixth People’s Hospital, Shanghai Jiao Tong University School of Medicine, Shanghai 200233, China; 4 Department of Obstetrics and Gynecology, University of Texas Southwestern Medical Center, Dallas, TX 75390, USA; jayanthi.lea@utsouthwestern.edu; 5 Harold C. Simmons Comprehensive Cancer Center, University of Texas Southwestern Medical Center, Dallas, TX 75390, USA; 6 Department of Pathology, Obstetrics and Gynecology Hospital of Tongji University, Shanghai 200092, China; zht1115@tongji.edu.cn

**Keywords:** estrogen receptor–negative (ER-negative) endometrial carcinomas, endometrial gastrointestinal-type adenocarcinoma, pilomatrix-like high-grade endometrial carcinoma, PiMHEC, mesonephric-like adenocarcinoma

## Abstract

Estrogen receptor–negative (ER-negative) endometrial carcinomas represent an emerging and historically underrecognized diagnostic concept encompassing a biologically aggressive and heterogeneous subset of endometrial cancers. Although loss of ER expression is increasingly recognized as an adverse prognostic indicator, ER negativity alone is insufficient for precise classification and must be interpreted within a histotype-specific and molecularly informed context. In this commentary, we review the evolving role of ER negativity in endometrial carcinoma through an integrated morphologic, immunophenotypic, and molecular framework, emphasizing both pathogenetic insights and practical diagnostic considerations. We highlight specific high-grade ER-negative tumor entities that merit particular diagnostic attention, including endometrial gastrointestinal-type adenocarcinoma, pilomatrix-like high-grade endometrial carcinoma, mesonephric-like adenocarcinoma, endometrial clear cell carcinoma, and ER-negative high-grade carcinomas not otherwise specified. These tumors exhibit distinct morphologic features and marked molecular heterogeneity that cannot be captured by hormone receptor status alone. Key diagnostic clues and common pitfalls are discussed, underscoring a practical workflow in which ER negativity serves as a diagnostic signal rather than a terminal category. Improved recognition and subclassification of ER-negative endometrial carcinomas are essential for accurate diagnosis, prognostic stratification, and optimized clinical management.

## Simple Summary

Estrogen receptor–negative (ER-negative) endometrial carcinomas represent a biologically aggressive and heterogeneous subset of endometrial cancers. Although ER testing has long been used in endometrial carcinoma, it has historically been applied mainly for therapeutic decision-making rather than as a diagnostic tool. Loss of ER expression is associated with poor prognosis but, by itself, is insufficient for accurate tumor classification. In this commentary, we review the evolving diagnostic significance of ER negativity using an integrated framework that incorporates tumor morphology, immunophenotypic features, and molecular heterogeneity. We highlight several high-grade ER-negative tumor types—including gastrointestinal-type adenocarcinoma, pilomatrix-like carcinoma, mesonephric-like adenocarcinoma, clear cell carcinoma, and other high-grade ER-negative carcinomas—that show distinct clinicopathologic characteristics. We propose that ER negativity should be regarded as a diagnostic signal that prompts careful subclassification, with important implications for accurate diagnosis and clinical management.

## 1. Introduction

Historically, estrogen receptor (ER) assessment in endometrial carcinoma (EC) has been viewed largely through a therapeutic lens, primarily to identify candidates for hormonal treatment, rather than as a marker of intrinsic tumor biology. Consequently, loss of ER expression has often been regarded as a secondary or ancillary finding and frequently subsumed within broad histologic categories, most notably uterine serous carcinoma. This pragmatic but simplified approach has become increasingly inadequate in light of the expanding recognition of biological and clinical heterogeneity in endometrial cancer.

The incorporation of molecular classification into routine diagnostic practice has further underscored the limitations of histotype-based generalization. ER-negative tumors are not restricted to a single histologic subtype or molecular category but occur across diverse diagnostic contexts. Emerging evidence indicates that ER negativity is associated with aggressive tumor behavior, including within molecularly defined subgroups traditionally considered to carry intermediate risk. These observations suggest that ER loss conveys biologic and diagnostic significance that extends well beyond its role in therapeutic decision-making.

In this commentary review, we propose a reframing of ER-negative endometrial carcinoma (ERnegEC) as a diagnostic category that merits deliberate histotype-level subclassification. ER-negative endometrial carcinomas are intrinsically heterogeneous, and ER negativity should be viewed as a diagnostic cue that prompts careful consideration of specific entities—such as mesonephric-like adenocarcinoma—that may otherwise be underrecognized. By integrating morphologic, immunophenotypic, and molecular perspectives, we aim to define the diagnostic and clinical implications of ER negativity and to propose a practical framework that supports informed clinical decision-making.

## 2. Defining ER Negativity in Endometrial Carcinoma

Despite the widespread use of estrogen receptor (ER) immunohistochemistry in endometrial carcinoma (EC), there is currently no universally accepted, outcome-based consensus regarding the optimal threshold for defining ER negativity. In routine practice, applied cutoffs vary by clinical context and intended use and are largely extrapolated from breast cancer paradigms, with many studies adopting a threshold of <1% ER expression [1,2]. However, emerging outcome-driven data suggest that this binary approach may be overly restrictive in EC.

A large European multicenter study systematically evaluated a broad range of ER expression thresholds and demonstrated that tumors exhibiting 0–10% ER expression consistently showed adverse disease-specific and disease-free survival. These findings provide outcome-based support for adopting 10% as a pragmatic lower threshold to define ER negativity in EC [3]. From a practical standpoint, use of a 10% cutoff allows more consistent identification of tumors with biologically and clinically relevant loss of ER expression, facilitating harmonization across studies and diagnostic practice.

At the population level, ER negativity is unevenly distributed across endometrial carcinoma subtypes and frequently overlaps with other high-risk clinicopathologic and molecular features. Broadly, ER-negative endometrial carcinomas parallel the spectrum of invasive histologic tumor types recognized in the 2023 FIGO classification [3]. Conceptually, ER-negative tumors can be divided into two groups: (1) established endometrial carcinoma types that typically retain ER expression but include a minority of ER-negative cases, and (2) carcinoma subtypes in which ER negativity is a defining or near-universal feature.

Within the first group, low-grade endometrioid endometrial carcinoma (LG-EEC) generally retains ER expression, although a small subset of cases shows ER loss. ER negativity is more frequently encountered in high-grade endometrioid carcinomas and in endometrial serous carcinomas [4,5]. In contrast, ER negativity is a consistent feature in several distinct endometrial carcinoma subtypes, including endometrial clear cell carcinoma [6], mesonephric-like adenocarcinoma (MLA) [7], endometrial gastrointestinal-type adenocarcinoma (EmGA) [8,9], pilomatrix-like high-grade endometrial carcinoma (PiMHEC) [10], and undifferentiated/dedifferentiated endometrial carcinomas.

## 3. ER Negativity in the Context of Molecular Classification

Within the contemporary TCGA molecular framework, endometrial carcinomas are conventionally classified into four major categories: ultramutated tumors with polymerase epsilon (POLE) mutations, hypermutated tumors with microsatellite instability (MSI), copy number–high tumors characterized by TP53 mutations, and copy number–low (CNL) tumors, also referred to as no-specific molecular profile (NSMP) tumors [11,12,13]. The Proactive Molecular Risk Classifier for Endometrial Cancer (ProMisE) offers a clinically practical surrogate for TCGA classification by incorporating widely available immunohistochemical markers, using mismatch repair (MMR) deficiency as a proxy for MSI and p53 immunohistochemistry as a surrogate for TP53 mutation status [14]. This pragmatic molecular framework has represented a major advance in risk stratification and has been widely adopted in clinical trials and international guidelines.

Despite these advances, NSMP tumors account for approximately half of all endometrial carcinomas and remain the most heterogeneous molecular category, encompassing lesions with markedly divergent histology, biology, and clinical behavior [15]. This intrinsic heterogeneity highlights a structural limitation of current molecular classification schemes and underscores the need for additional biomarkers that are low-cost, reproducible, and broadly accessible. Within this context, ER expression has emerged as a clinically informative modifier of molecular risk in endometrial cancer.

Detailed analyses of ER expression patterns within NSMP endometrial carcinomas have shown that the majority of tumors exhibit uniformly high ER expression and correspond to conventional endometrioid histology and good prognosis. In contrast, ER-negative tumors represent a biologically and morphologically distinct minority that is disproportionately enriched for non-endometrioid carcinoma types, including clear cell carcinoma, mesonephric-like adenocarcinoma, gastric-type adenocarcinoma–related tumors, carcinosarcoma, and unclassifiable high-grade carcinomas, as well as a subset of high-grade endometrioid carcinomas [16,17]. These findings reinforce that ER negativity should not be regarded as a passive ancillary observation, but rather as a diagnostic signal that warrants careful morphologic reassessment and a targeted immunohistochemical workup.

Emerging evidence further supports the clinical utility of incorporating ER status into contemporary molecular classification to refine risk stratification. Specifically, subdivision of the NSMP category into ER-positive and ER-negative subsets identifies clinically meaningful differences in outcome [17]. In large cohort studies by Vermij and colleagues, NSMP tumors retaining ER expression were associated with favorable recurrence-free survival, comparable to intermediate-risk groups, whereas NSMP tumors with ER loss demonstrated significantly inferior outcomes that approached or overlapped those observed in p53-abnormal carcinomas [18]. Collectively, these data support a pragmatic five-tier risk stratification framework comprising POLEmut, NSMP–ERpos, MMRd, p53abn, and NSMP–ERneg tumors, ordered from most favorable to most adverse prognosis. Importantly, this framework does not redefine established molecular subtypes but instead provides a functional refinement that integrates molecular classification with hormonal status to address residual heterogeneity within the NSMP group.

POLE exonuclease-domain–mutated endometrial carcinomas represent a distinct molecular subgroup characterized by ultramutation, excellent prognosis, and very low recurrence risk, even in tumors with high-grade histologic features. Current ESGO/ESTRO/ESP guidelines recognize their favorable biology and support treatment de-escalation in early-stage disease [19,20]. Importantly, POLE-mutated tumors are overwhelmingly endometrioid in type and typically retain ER expression [21], underscoring that the ultramutated pathway is largely restricted to hormonally differentiated carcinomas and is rarely encountered in ER-negative endometrial cancers. This distinction further highlights the biological divergence between POLE-mutated tumors and the ER-negative entities emphasized in this review.

## 4. ER Negative Endometrial Cancer with Poor Prognosis

Although ER and progesterone receptor (PR) assessment in endometrial carcinoma has historically been viewed primarily through a therapeutic lens, accumulating evidence demonstrates that hormone receptor status retains independent prognostic value, even within TCGA/ProMisE-defined molecular subgroups. In a large multicenter cohort study (n = 739), Verde and colleagues classified tumors using next-generation sequencing and/or immunohistochemical surrogates into POLEmut (9.1%), MMRd (27.6%), p53mut (20.8%), and NSMP (42.5%) categories and showed that a three-tiered ER/PR stratification (0–10%, 20–80%, 90–100%) provided additional prognostic discrimination across molecular classess [22]. Notably, ER expression refined outcome prediction within both MMRd and NSMP tumors, with ER 0–10% NSMP carcinomas demonstrating substantially inferior 5-year disease-specific survival compared with ER-high counterparts, supporting the concept that hormone receptor loss identifies a biologically aggressive subset not fully captured by molecular classification alone [22].

Complementary findings were reported by Jamieson and colleagues in a large cohort of 1110 NSMP endometrial carcinomas [2]. In that study, tumor grade and ER expression emerged as the strongest prognostic variables within the NSMP category, enabling stratification into a low-risk group (FIGO grade 1–2, ER-positive) with very low disease-specific mortality and a high-risk group defined by FIGO grade 3 and/or ER-negative status. Importantly, ER-negative NSMP tumors accounted for the majority of NSMP-related deaths and exhibited clinical outcomes approaching those of established aggressive histologic subtypes. ER loss was independently associated with adverse disease-specific and progression-free survival and was enriched among clear cell, dedifferentiated, and mesonephric-like carcinomas [2].

Consistent observations have also emerged from randomized trial cohorts. In the PORTEC studies, ER expression assessed using a pragmatic 10% cutoff was associated with increased risk of recurrence and inferior survival in patients with early-stage endometrial carcinoma on univariable analysis [23]. Although the prognostic impact of ER loss partially overlapped with established molecular and clinicopathologic risk factors, these data further support the concept that ER negativity reflects adverse tumor biology. More recently, clinicopathologic and molecular characterization of high-grade endometrioid carcinomas demonstrated that ER-negative NSMP tumors were associated with particularly poor clinical outcomes, adding to the growing body of evidence supporting ER loss as a high-risk feature within the NSMP group [17].

## 5. ER Negative Endometrial Carcinoma Subclassification

As outlined above, ER-negative endometrial carcinomas are consistently associated with adverse clinical outcomes, particularly within the NSMP molecular category. However, ER negativity alone does not define a single disease entity. Instead, it encompasses a spectrum of biologically and morphologically distinct tumors with different histogenesis, molecular profiles, and clinical implications.

In routine diagnostic practice, accurate classification and appropriate clinical management therefore depend on correct histologic subclassification rather than hormone receptor status alone. In this context, ER negativity should be regarded as a diagnostic entry point that prompts systematic reassessment of tumor morphology and judicious use of targeted immunohistochemical and molecular studies, rather than as a terminal diagnostic label.

ER-negative endometrial carcinomas comprise multiple distinct histologic entities, including clear cell carcinoma (>90% ER negativity) [24], mesonephric-like adenocarcinoma (approximately 75–100% ER-negativity) [7], endometrial gastrointestinal-type adenocarcinoma (>90% ER-negativity) [25], pilomatrix-like high-grade endometrial carcinoma (PiMHEC, >90% ER-negativity) [26], and ER-negative high-grade endometrial carcinoma, not otherwise specified (ERneg HGEC-NOS), which typically demonstrates near-complete loss of ER expression.

In addition, ER negativity may also be encountered in the differential diagnosis, particularly in cervical adenocarcinomas (approximately 60% ER negativity) [27,28], as well as in a subset of low-grade endometrioid carcinomas (4–7% ER negativity), high-grade endometrioid carcinomas (18–53% ER negativity), mucinous carcinomas (50–92% ER negativity) [4], and undifferentiated carcinomas (>90% ER negativity) [29,30]. Accurate subclassification therefore requires careful integration of morphologic features, immunophenotypic profiles, and clinical context to distinguish true ER-negative endometrial carcinoma subtypes from morphologic and immunophenotypic mimics.

## 6. Specific High-Grade ER-Negative Endometrial Carcinomas Deserve Diagnostic Attention

In current practice, identification of ER-negative endometrial carcinomas begins with routine estrogen receptor immunohistochemistry, which is now performed universally for endometrial cancers in most pathology laboratories in the United States and Europe. This practice provides a practical and reproducible foundation for recognizing tumors with loss of ER expression and serves as the initial step in identifying clinically relevant ER-negative carcinomas.

As outlined above, ER negativity is associated with adverse clinical outcomes and refines risk stratification within current molecular frameworks, particularly in the NSMP category. However, the diagnostic significance of ER loss extends beyond prognostication. In routine diagnostic settings, ER negativity should be regarded as an early diagnostic signal rather than a terminal diagnosis, prompting focused histologic reassessment and consideration of specific high-grade carcinoma subtypes with distinct morphologic, immunophenotypic, and biologic features.

In this section, we focus on selected high-grade ER-negative endometrial carcinoma subtypes in which ER loss is a defining or near-universal feature and which therefore merit particular diagnostic attention. The discussion emphasizes key recognition features and common diagnostic pitfalls rather than exhaustive characterization. Detailed diagnostic pearls and practical algorithms are addressed in subsequent sections.

### 6.1. Endometrial Gastrointestinal-Type Adenocarcinoma (EmGA)

Endometrial gastrointestinal-type adenocarcinoma (EmGA) is a rare but aggressive subtype of endometrial carcinoma that remains diagnostically challenging and is frequently underrecognized. It predominantly affects postmenopausal women and typically presents with abnormal uterine bleeding. The true incidence is difficult to ascertain because of its rarity and historical misclassification [8,31]. Although the term “gastric-type” adenocarcinoma has been used historically, “gastrointestinal-type” is more accurate, as many tumors show intestinal differentiation with identifiable goblet cells [32]. Since its formal inclusion in the 5th edition of the World Health Organization (WHO) Classification of Female Genital Tumors in 2020 [33], increasing awareness has led to a growing number of reported cases and clinicopathologic studies [9,31].

**Figure 1:**
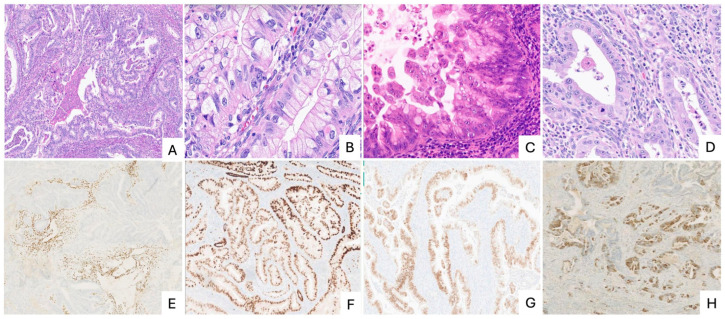
This Endometrial gastrointestinal mucinous adenocarcinoma (EmGA). (**A**) Low-power view showing a predominantly glandular growth pattern; (**B**) High-power view demonstrating tumor cells with abundant eosinophilic to clear cytoplasm and basally located nuclei; (**C**) Scattered intestinal-type goblet cells are present; (**D**) Areas lacking overt gastrointestinal-type differentiation are also identified within the tumor; (**E**) Tumor cells are negative for estrogen receptor (ER); (**F**) Tumor cells show retained PAX8 expression; (**G**) Nuclear positivity for CDX2 supports gastrointestinal differentiation; (**H**) Claudin 18 demonstrates membranous positivity, consistent with gastric-type differentiation.

The diagnosis of EmGA rests on the identification of mucinous glands with gastric or gastrointestinal-type morphology together with at least focal expression of one or more gastrointestinal immunohistochemical markers (Figure 1). Estrogen receptor expression is absent or minimal. By definition, there should be no conventional endometrioid carcinoma component, no evidence of an extrauterine primary tumor, and no dominant cervical involvement. When these histologic and immunophenotypic features are well developed, diagnosis is usually straightforward. However, EmGA frequently poses diagnostic challenges because of substantial morphologic and immunohistochemical overlap with other endometrial and cervical carcinomas, as well as with metastatic tumors from the gastrointestinal or pancreaticobiliary tract.

Conventional low-grade endometrioid carcinoma with mucinous differentiation is a common differential diagnostic consideration but rarely causes difficulty, as it typically retains ER expression. In contrast, cervical adenocarcinoma—both HPV-associated and HPV-independent (gastric-type)—represents a more significant diagnostic challenge when intestinal differentiation is present. Intestinal-type goblet cells are common in HPV-associated cervical adenocarcinoma. Features favoring a cervical primary include a cervical-centered tumor, the presence of endocervical precursor lesions such as adenocarcinoma in situ (usual-type or gastric-type) or lobular endocervical glandular hyperplasia (LEGH), and detection of high-risk HPV in tumor tissue, as EmGA is not HPV-associated and typically lacks identifiable precursor lesions. Although EmGA may secondarily extend into the endocervix, assignment of an endometrial primary relies primarily on the dominant tumor location, as immunophenotypic profiles substantially overlap between EmGA and cervical gastric-type adenocarcinoma.

Exclusion of metastatic mucinous carcinoma from the gastrointestinal or pancreaticobiliary tract is also essential. This requires careful correlation with clinical history and radiologic findings, as morphology and immunohistochemistry alone are often insufficient due to significant overlap with primary gastrointestinal tumors. PAX8 expression may support a gynecologic origin; however, approximately one third of EmGAs are PAX8 negative, limiting its sensitivity [8].

A particularly challenging diagnostic scenario arises when EmGA shows marked morphologic heterogeneity or high-grade cytology without overt gastrointestinal differentiation. In such cases, tumors are frequently misclassified as endometrial serous carcinoma or poorly differentiated carcinoma, especially given the frequent presence of abnormal p53 expression [9,31,34]. In this setting, it is critical to consider EmGA in the differential diagnosis of ER-negative endometrial carcinomas and to apply a focused panel of gastrointestinal markers. At least one marker—such as CK20, CDX2, SATB2, MUC6, HIK1083, or Claudin 18—is typically positive in EmGA and can often resolve the diagnostic dilemma [8,34].

Endometrial clear cell carcinoma represents another important morphologic mimic, as tumor cells may display abundant clear cytoplasm and lack ER and PR expression. However, clear cell carcinoma lacks true intracytoplasmic mucin and is negative on mucin stains. Identification of characteristic architectural features, such as tubulopapillary growth, hobnail cells and stromal hyalization, together with immunoreactivity for Napsin A, HNF1β, and AMACR, and absence of gastrointestinal marker expression, supports a diagnosis of clear cell carcinoma rather than EmGA [8,34].

Accurate recognition of EmGA has important clinical implications. Claudin 18, a marker of gastric-type differentiation, is increasingly being explored as a potential therapeutic target, further underscoring the need for precise diagnosis of this aggressive and clinically relevant tumor subtype [31,35].

### 6.2. Pilomatrix-like High-Grade Endometrial Carcinoma (PiMHEC)

Pilomatrix-like high-grade endometrial carcinoma (PiMHEC) is a recently recognized and highly aggressive subtype of endometrial carcinoma affecting a wide age range. Its clinical course is particularly unfavorable, with reported outcomes worse than those of conventional endometrial serous carcinoma [36,37]. To avoid conceptual confusion with endometrioid carcinoma, recent studies favor the term endometrial rather than endometrioid, while retaining the acronym PiMHEC.

**Figure 2:**
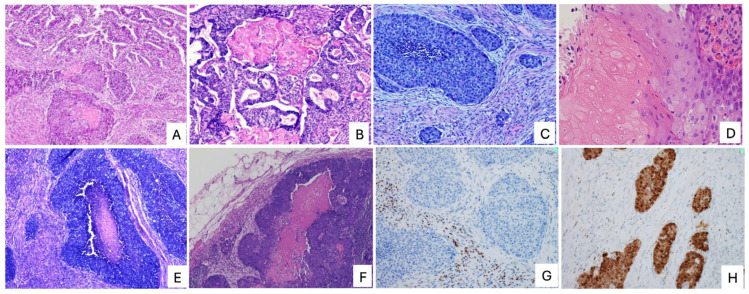
Pilomatrix-like high-grade endometrial carcinoma (PiMHEC). (**A**) Dual architectures with a superficial low-grade component (upper portion) and an underlying high-grade component (lower portion); (**B**) Shadow cells (upper middle) associated with the low-grade component; (**C**) High-grade basaloid component with extensive lymphovascular space invasion (LVSI); (**D**) Shadow cells (left) admixed with areas of squamous metaplasia (right); (**E**) Basaloid tumor nests exhibiting characteristic comedo-type necrosis; (**F**) Lymph node metastasis composed exclusively of the high-grade component; (**G**) Complete loss of ER expression in tumor cells; (**H**) Diffuse aberrant β-catenin expression involving both nuclei and cytoplasm.

Histologically, PiMHEC is characterized by a biphasic pattern composed of admixed low-grade and high-grade components. The high-grade component consists of nests and sheets of basaloid tumor cells with extensive tumor necrosis, often in a geographic or comedo-like pattern. Shadow cells, reminiscent of those seen in cutaneous pilomatrix carcinoma, represent a defining morphologic feature and may be focal, particularly in biopsy specimens [37,38,39]. Metastatic deposits are typically composed predominantly or exclusively of the high-grade component (Figure 2), underscoring its role in driving aggressive clinical behavior.

Immunophenotypically, the high-grade component of PiMHEC is uniformly ER negative and shows diffuse aberrant nuclear and cytoplasmic β-catenin expression with a p53 wild-type staining pattern [10]. Limited expression of neuroendocrine markers is common, and focal expression of gastrointestinal markers such as CDX2 or SATB2 may be observed, representing potential diagnostic pitfalls [10,37,40,41]. At the molecular level, most tumors fall within the NSMP category, with a subset demonstrating mismatch repair deficiency; POLE-mutated and p53-abnormal molecular profiles have not been documented to date [38].

The most frequent diagnostic mimic of PiMHEC is FIGO grade 3 endometrioid carcinoma, as both entities may show admixed low- and high-grade components. However, grade 3 endometrioid carcinoma typically demonstrates solid or cribriform architecture with variable nuclear atypia, often retains ER expression, exhibits limited tumor necrosis, and shows absent or focal nuclear β-catenin staining. In contrast, PiMHEC displays basaloid tumor nests with uniformly high-grade cytology, complete loss of ER and PR expression, extensive geographic or comedo-like necrosis, diffuse nuclear β-catenin accumulation, and characteristic shadow cells. Recognition of this constellation of features is critical for accurate diagnosis.

Dedifferentiated endometrial carcinoma represents another important consideration, particularly because it also exhibits biphasic morphology and hormone receptor loss in the high-grade component. However, the undifferentiated component of dedifferentiated carcinoma lacks epithelial differentiation, showing loss of BRG1 expression and minimal or absent cytokeratin and β-catenin staining, without shadow cells. In contrast, PiMHEC retains epithelial markers and shows diffuse nuclear β-catenin staining, allowing reliable distinction between these entities.

Other potential mimics include neuroendocrine carcinoma and poorly differentiated squamous cell carcinoma. Neuroendocrine marker expression in PiMHEC is typically focal and limited, falling below the threshold required for a diagnosis of neuroendocrine carcinoma [40] and should be regarded as incidental. Poorly differentiated squamous cell carcinoma may be considered because of the basaloid appearance of the high-grade component; however, primary endometrial squamous cell carcinoma is rare, and metastatic squamous cell carcinoma from the lower genital tract can usually be excluded based on clinical history and identification of a primary lesion. Importantly, shadow cells and comedo-type necrosis are not features of squamous cell carcinoma and strongly favor a diagnosis of PiMHEC.

The pathogenesis of PiMHEC remains incompletely understood. Based on our recent study [10] and prior reports [36,42,43,44,45,46,47,48], CTNNB1 exon 3 mutation appears to be a distinctive and recurrent molecular feature. The identification of identical CTNNB1 mutations in spatially distinct low-grade and high-grade components supports a clonal relationship and suggests progression through dedifferentiation from a low-grade endometrioid precursor. However, CTNNB1 mutations are also observed in other gynecologic lesions, including morular metaplasia in atypical endometrial hyperplasia and subsets of conventional endometrioid carcinomas [49], indicating that this alteration alone is insufficient to explain the extreme aggressiveness of PiMHEC. Additional mutations involving ARID1A, PTEN, and PIK3CA have been identified but likewise fail to fully account for its clinical behavior. Notably, TP53 mutations have not been identified in PiMHEC, despite its paradoxically poorer prognosis compared with endometrial serous carcinoma. These observations underscore the molecular complexity of PiMHEC and highlight the need for further studies to elucidate its pathogenesis and identify targetable biomarkers.

### 6.3. Mesonephric-like Adenocarcinoma (MLA)

Mesonephric-like adenocarcinoma (MLA) of the endometrium is a rare and clinically aggressive carcinoma, accounting for approximately 1% of endometrial cancers. It occurs across a wide age range, from the third to the eighth decade of life, and is associated with an increased risk of early recurrence and distant metastasis [50].

MLA exhibits striking morphologic heterogeneity and may display a wide spectrum of architectural patterns, including tubular, ductal, papillary, retiform, glomeruloid, sieve-like, corded, solid, or spindle cell growth. Among these, small tubular and ductal/glandular patterns are most common [51]. Tumor nuclei are typically relatively bland, with mild atypia, angulated or oval contours, and dense or vesicular chromatin that may resemble papillary thyroid carcinoma–like nuclear features. Hobnail cells may be present, creating overlap with clear cell carcinoma. Glandular lumina often contain brightly eosinophilic intraluminal secretions, although this feature is neither universal nor specific and may also be encountered in endometrioid carcinomas.

Because of its morphologic variability and relatively bland cytology, MLA has historically been misdiagnosed as other, more common Müllerian tumors. Low-grade endometrioid carcinoma is the most frequent diagnostic pitfall, particularly when MLA shows predominantly glandular growth [52]. Cases with prominent stromal hyalinization may be misinterpreted as corded and hyalinized endometrioid carcinoma [53]. In other settings, MLA may mimic high-grade carcinomas, including clear cell carcinoma, serous carcinoma, or even carcinosarcoma. Occasional admixture of MLA with conventional Müllerian carcinomas further supports a Müllerian origin rather than derivation from true mesonephric remnants [54].

**Figure 3:**
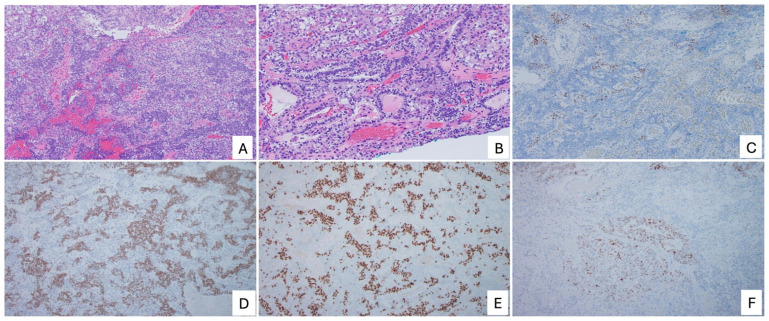
Endometrial mesonephric-like adenocarcinoma (MLA). (**A**) Low-power view showing a trabecular growth pattern; (**B**) Intermediate-power view demonstrating columnar tumor cells with clear cytoplasm; (**C**) Tumor cells are negative for ER; (**D**) GATA3 and (**E**) TTF-1 immunostains performed on the same field as panel A, demonstrating a reciprocal staining pattern; (**F**) Wild-type p53 expression pattern.

**Figure 4:**
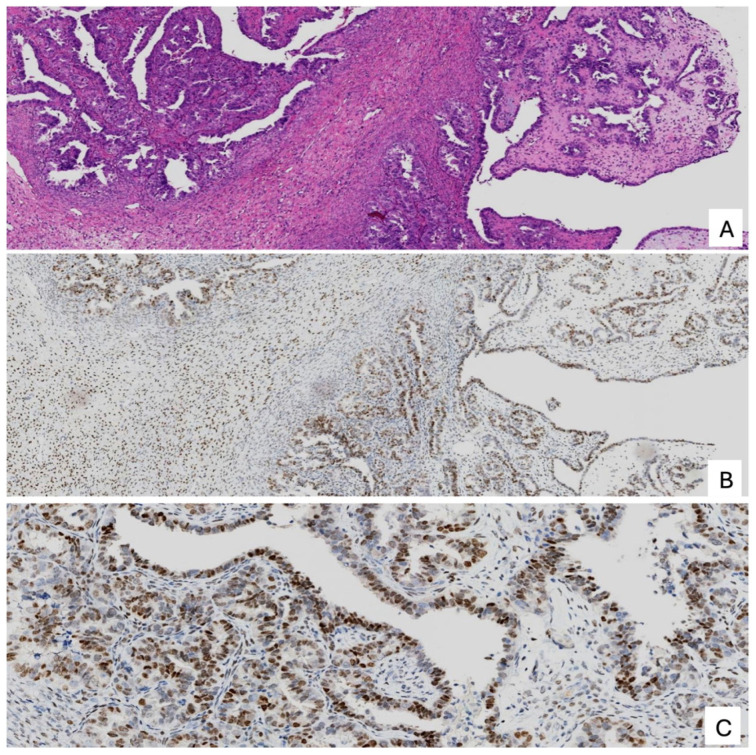
Mesonephric-like adenocarcinoma with retained ER expression (>10%). (**A**) Hematoxylin and eosin–stained section showing representative MLA morphology (corresponding GATA3 and TTF-1 positivity not shown); (**B**) ER immunostain at the same magnification as panel A demonstrating approximately 70% nuclear positivity; (**C**) Higher-power view highlighting diffuse ER expression in tumor nuclei.

Immunohistochemically, MLA typically shows expression of PAX8 and variable positivity for GATA3 and TTF-1, often in a reciprocal pattern (Figure 3). Hormone receptor expression is usually absent; however, ER positivity exceeding 10% of tumor nuclei has been reported in a subset of cases and does not exclude the diagnosis [54]. Importantly, when the morphologic features and overall immunophenotypic profile are characteristic of MLA, the diagnosis should be maintained even in the presence of ER expression above 10% (Figure 4). CD10 may show luminal or focal staining, although this finding is inconsistent across studies [49,55]. p53 shows a wild-type pattern, mismatch repair protein expression is retained, and p16 typically demonstrates a non-block (mosaic) staining pattern. When morphologic features are atypical, integration of p53 and MMR immunostaining with additional markers such as CD10, PAX2, and SOX17 may be helpful. PAX8 expression is expected in MLA, and lack of diffuse PAX8 staining should prompt reconsideration of the diagnosis. SOX17 negativity has been proposed as a supportive feature distinguishing MLA from other Müllerian tumors, although this marker is not uniformly negative in all cases [56]. Deviations from the expected immunophenotypic profile should raise the possibility of an alternative diagnosis or, in the presence of distinct morphologic and immunohistochemical patterns, a mixed carcinoma.

At the molecular level, most MLAs harbor activating KRAS mutations and fall within the NSMP molecular category with a p53 wild-type profile [57]. Although targeted inhibitors of mutant KRAS have shown promise in other solid tumors [58], only a small subset of endometrial MLAs harbor targetable KRAS p.G12C alterations. Current data suggest that approximately 8% of MLAs may be theoretically eligible for such therapy, limiting its immediate clinical applicability [59]. These findings reinforce that accurate diagnosis of MLA relies primarily on careful integration of morphologic and immunophenotypic features, with molecular findings serving a complementary role.

### 6.4. Endometrial Clear Cell Carcinoma (ECCC)

Endometrial clear cell carcinoma (ECCC) is a well-established estrogen receptor–negative subtype of endometrial carcinoma, accounting for less than 5% of all cases. It occurs predominantly in older women and appears to be more prevalent in East Asian populations. Unlike conventional endometrioid carcinoma, ECCC is not associated with estrogen stimulation and typically demonstrates intrinsically aggressive clinical behavior, with the majority of tumors behaving as high-risk disease [55].

Morphologically, ECCC exhibits a characteristic but variable spectrum of architectural and cytologic features. Tumors may display solid, tubular/glandular, papillary, or mixed growth patterns. Stromal hyalinization may be present but is uncommon and not required for diagnosis. Cytologically, tumor cells are polygonal with moderate to abundant clear or eosinophilic cytoplasm and are usually arranged in a single cell layer. Hobnail and flat cells are frequently observed. Nuclear atypia is variable, with occasional enlarged or irregular nucleoli, and mitotic activity is often relatively low. Importantly, the presence of a high mitotic index or marked nuclear pleomorphism does not exclude a diagnosis of ECCC when other prototypical features are present. Additional findings such as targetoid bodies, eosinophilic intracytoplasmic globules, or psammoma bodies may be encountered in a subset of cases.

Diagnosis of ECCC should be grounded in recognition of its characteristic architectural and cytologic features. When classic morphology is present, diagnosis is usually straightforward. However, ECCC may exhibit unusual or overlapping features, particularly in limited biopsy specimens, underscoring the importance of careful morphologic evaluation supported by targeted immunohistochemistry.

Immunohistochemically, ECCC typically expresses PAX8 and CK7. HNF-1β is the most sensitive marker, showing positivity in approximately 67–100% of cases, although it lacks specificity. Napsin A (56–93%) and AMACR (75–88%) are also commonly positive, with Napsin A considered relatively more specific among these markers. Mismatch repair (MMR) protein expression is retained in approximately 80–90% of cases. Hormone receptors are typically negative.

From a molecular perspective, ECCC spans all four TCGA molecular categories, with enrichment in p53 wild-type and p53-abnormal subtypes and rare POLE-mutated cases [56,57]. Importantly, molecular subclassification has prognostic significance: ECCC with mismatch repair deficiency demonstrates clinical behavior comparable to p53-abnormal tumors and is associated with poor outcomes [57]. In contrast, the rare POLE-mutated ECCC cases may show a more favorable prognosis. These observations highlight that clinical outcome in ECCC correlates more closely with molecular subclassification than with histologic grade alone, emphasizing the value of integrated morphologic and molecular assessment [57,58].

Given its long-standing recognition, the differential diagnosis of ECCC is only briefly summarized here. Key considerations include ovarian clear cell carcinoma, in which endometrial involvement supports an endometrial primary; endometrial serous carcinoma, particularly when p53 is abnormal, unless classic clear cell morphology is present; and endometrioid carcinoma with clear cell or secretory change, which typically shows low-grade nuclear features, associated atypical hyperplasia, and strong, diffuse ER expression. Arias–Stella reaction should be considered in younger patients or those with pregnancy or progestin exposure and is characterized by preserved glandular architecture and low proliferative activity. Clear cell carcinoma of cervical origin cannot be reliably distinguished morphologically and requires correlation with tumor location. Metastatic renal cell carcinoma, clear cell type, should be excluded based on clinical history and immunoprofile, as these tumors are typically CK7-, ER-, and PR-negative but positive for CD10 and HNF-1β.

Therapeutic strategies for ECCC increasingly focus on molecular alterations rather than histologic subtype alone. Current approaches include immune checkpoint inhibitors such as dostarlimab and pembrolizumab, VEGFR inhibitors such as lenvatinib, often in combination with platinum-based chemotherapy for advanced or recurrent disease, and ongoing investigation of PARP inhibitors and HER2-targeted therapies in selected molecular contexts [59]. These advances further underscore the importance of accurate diagnosis and molecular characterization in guiding clinical management of this aggressive endometrial carcinoma subtype.

### 6.5. ER-Negative Endometrial Carcinoma, Not Otherwise Specified (ERneg EC-NOS)

**Figure 5:**
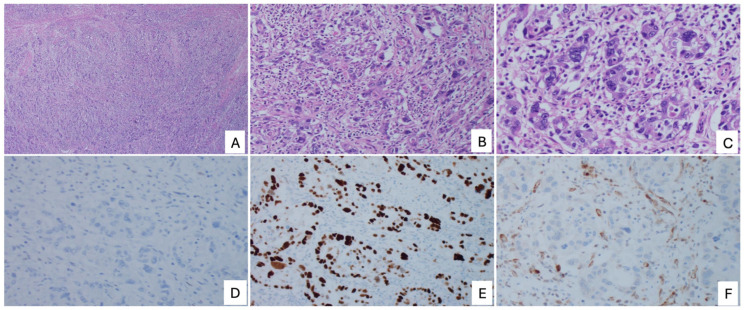
ER-negative high-grade endometrial carcinoma, not otherwise specified (ERneg EC-NOS). (**A**) Low-power view showing a predominantly solid growth pattern; (**B**) Intermediate-power view demonstrating marked nuclear atypia with bizarre tumor nuclei; (**C**) High-power view further illustrating pronounced nuclear pleomorphism; (**D**) Complete loss of ER expression; (**E**) Aberrant (mutant-type) p53 immunostaining pattern; (**F**) Loss of PTEN expression in tumor cells.

After exclusion of all recognized ER-negative endometrial carcinoma subtypes, a small but clinically significant subset of high-grade tumors remains that cannot be confidently assigned to a defined histologic category. These tumors are designated ER-negative endometrial carcinoma, not otherwise specified (ERneg EC-NOS). By definition, this is a diagnosis of exclusion and represents a heterogeneous and incompletely characterized group. Morphologically, many cases overlap with poorly differentiated or undifferentiated carcinoma, although they lack sufficient defining features for definitive classification (Figure 5).

Limited molecular data suggest that ERneg EC-NOS cases are distributed primarily across the NSMP and p53-abnormal molecular categories; however, no consistent or pathognomonic molecular alterations have been identified. It remains unclear whether ERneg EC-NOS represents a truly heterogeneous collection of biologically unrelated tumors or, alternatively, encompasses additional distinct entities that have yet to be recognized. Further systematic clinicopathologic and molecular studies will be required to better define this category and to clarify its diagnostic boundaries and clinical significance.

## 7. Conclusions

ER-negative endometrial carcinomas comprise a heterogeneous group of tumors that cannot be adequately defined by hormone receptor status alone. Accurate diagnosis relies on careful histotype-level evaluation, with morphology as the foundation and immunohistochemistry and molecular data applied in a targeted, context-dependent manner. ER-negative tumors span multiple TCGA molecular categories and include several distinct, often underrecognized entities with important prognostic and therapeutic implications. Importantly, ER negativity should be regarded as a diagnostic cue rather than a final classification. An integrated morphologic, immunophenotypic, and molecular approach is essential to improve diagnostic precision, refine risk stratification, and guide contemporary clinical management.

## Data Availability

All the data has been shared in this publication.

## Abbreviations

The following abbreviations are used in this manuscript:

ER	Estrogen receptor
EC	Endometrial carcinoma
ERnegEC	Estrogen receptor–negative endometrial carcinoma
LG-EEC	Low-grade endometrioid endometrial carcinoma
MLA	Mesonephric-like adenocarcinoma
EmGA	Endometrial gastrointestinal-type adenocarcinoma
PiMHEC	Pilomatrix-like high-grade endometrial carcinoma
ERneg HGEC-NOS	Estrogen receptor–negative high-grade endometrial carcinoma, not otherwise specified
TCGA	The Cancer Genome Atlas
POLE	DNA polymerase epsilon
POLEmut	POLE-mutated
MSI	Microsatellite instability
MMR	Mismatch repair
MMRd	Mismatch repair–deficient
NSMP	No specific molecular profile
CNL	Copy number–low
p53abn	p53-abnormal
ProMisE	Proactive Molecular Risk Classifier for Endometrial Cancer
PR	Progesterone receptor
FIGO	International Federation of Gynecology and Obstetrics
WHO	World Health Organization
HPV	Human papillomavirus
LEGH	Lobular endocervical glandular hyperplasia
